# Overcoming Resistance to Selective Serotonin Reuptake Inhibitors: Targeting Serotonin, Serotonin-1A Receptors and Adult Neuroplasticity

**DOI:** 10.3389/fnins.2019.00404

**Published:** 2019-04-30

**Authors:** Faranak Vahid-Ansari, Min Zhang, Amin Zahrai, Paul R. Albert

**Affiliations:** Brain and Mind Research Institute, Ottawa Hospital Research Institute (Neuroscience), University of Ottawa, Ottawa, ON, Canada

**Keywords:** serotonin, antidepressant, autoreceptor, knockout, imaging, brain stimulation, noradrenalin, brain-derived growth factor

## Abstract

Major depressive disorder (MDD) is the most prevalent mental illness contributing to global disease burden. Selective serotonin (5-HT) reuptake inhibitors (SSRIs) are the first-line treatment for MDD, but are only fully effective in 30% of patients and require weeks before improvement may be seen. About 30% of SSRI-resistant patients may respond to augmentation or switching to another antidepressant, often selected by trial and error. Hence a better understanding of the causes of SSRI resistance is needed to provide models for optimizing treatment. Since SSRIs enhance 5-HT, in this review we discuss new findings on the circuitry, development and function of the 5-HT system in modulating behavior, and on how 5-HT neuronal activity is regulated. We focus on the 5-HT1A autoreceptor, which controls 5-HT activity, and the 5-HT1A heteroreceptor that mediates 5-HT actions. A series of mice models now implicate increased levels of 5-HT1A autoreceptors in SSRI resistance, and the requirement of hippocampal 5-HT1A heteroreceptor for neurogenic and behavioral response to SSRIs. We also present clinical data that show promise for identifying biomarkers of 5-HT activity, 5-HT1A regulation and regional changes in brain activity in MDD patients that may provide biomarkers for tailored interventions to overcome or bypass resistance to SSRI treatment. We identify a series of potential strategies including inhibiting 5-HT auto-inhibition, stimulating 5-HT1A heteroreceptors, other monoamine systems, or cortical stimulation to overcome SSRI resistance.

## Introduction

Major depressive disorder (MDD) has a high incidence and low remission rate with the current therapeutic strategies. Major depression is the largest contributor to global disability by years lived with disability, and anxiety disorders rank sixth (World Health Organization [WHO], 2017). The annual prevalence of depression is 4.4% overall, 3.6% in men and 5.1% in women (Baxter et al., 2014; World Health Organization [WHO], 2017). Major depression is diagnosed by persistent symptoms such as sadness, irritability, anhedonia or changes in appetite or sleep patterns that could result in suicidal thoughts and attempts (Kessler and Bromet, 2013). Genetic or biomarkers for major depression remain elusive, and current genome-wide association studies indicate that individual genetic polymorphisms contribute only a small increase in risk for depression. Brain imaging studies are beginning to reveal changes in functional connectivity associated with major depression that may predict treatment response (Drysdale et al., 2017; Dunlop et al., 2017). However, at present diagnosis of depression is made by psychiatric interviews, and treatment is not always effective.

Selective serotonin reuptake inhibitors (SSRIs) are the first-line treatment for major depression but are only effective for remission in 30% of patients (Rush et al., 2009). Furthermore, a latency of 2–3 weeks is required for response, and even longer to ascertain remission. Thus, a better understanding of how SSRIs mediate their actions could be useful to identify biomarkers or predictors of SSRI response and to enhance treatment efficacy.

**FIGURE 1:**
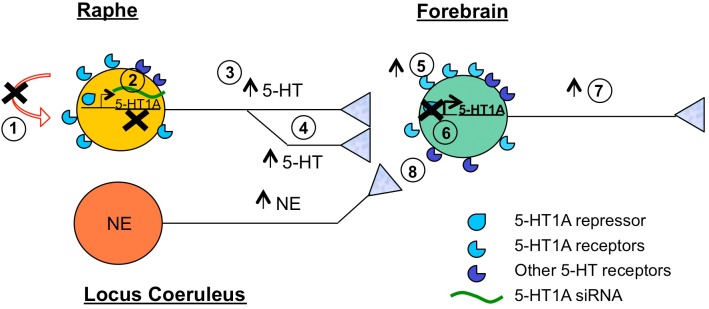
Summary of target sites to overcome SSRI resistance. Shown is a 5-HT neuron (yellow) that is refractory to SSRI treatment. Brain regions highlighted include raphe containing 5-HT neurons, locus coeruleus containing norepinephrine (NE) neurons (orange), and forebrain (green), which includes frontal cortex, hippocampus, amygdala, brain regions implicated in antidepressant response. Several potential sites of intervention to augment SSRI response or bypass SSRI treatment are shown. These include: (1) Blocking (X) auto-inhibition (via 5-HT1A autoreceptor or signaling blockers); (2) Reducing 5-HT1A autoreceptor expression (via desensitization, HTR1A gene repression or 5-HT-targeted 5-HT1A siRNA); (3) Increasing (arrow) 5-HT neuronal activity (via cortical stimulation); (4) Increasing 5-HT neuroplasticity (e.g., synaptogenesis); (5) Increasing 5-HT1A heteroreceptor activity (via biased 5-HT1A agonists); (6) Increasing 5-HT1A heteroreceptor expression (via inhibiting 5-HT1A-selective repressor activity); (7) Increasing cortical activity (via ketamine, cortical stimulation); (8) Bypassing 5-HT (via activation of other monoamines such as NE using transporter blockers or agonists).

SSRIs enhance the function of the serotonin (5-hydroxytryptamine, 5-HT) system and 5-HT has long been implicated as a mediator of antidepressant actions (Cowen, 2008). As a neuro-glial modulator, 5-HT functions throughout the body to regulate a diversity of homeostatic systems. In the brain, 5-HT is implicated in regulation of pain, sleep, appetite, stress, mood, and emotion (Jacobs and Azmitia, 1992). In this review, we discuss the actions of 5-HT in the nervous system and on behavior and how 5-HT activity is regulated, focusing on the 5-HT1A receptor, which both controls 5-HT activity and mediates 5-HT actions (Albert, 2012; Garcia-Garcia et al., 2014). We address how SSRI actions are mediated, mechanisms that promote resistance to chronic SSRI treatment, and how SSRI resistance may be predicted and overcome. In addition, while most animal studies were using males, we have noted studies that include females. Taken together, there is strong evidence from rodent models that increased 5-HT1A autoreceptor function contributes to depression and SSRI resistance, while activation of hippocampal 5-HT1A heteroreceptors is required for SSRI action. Several potential targets to bypass these mechanisms of SSRI resistance are highlighted including reducing 5-HT auto-inhibition, activating the 5-HT system pharmacologically or through brain stimulation, activating 5-HT1A heteroreceptors, or bypassing the 5-HT system by activating other monoamine systems (Figure 1).

In this review we focus mechanisms of resistance to SSRIs involving altered activity of 5-HT neurons via 5-HT1A autoreceptors and how these changes can be overcome by targeting 5-HT1A receptors, non-5-HT mechanisms or activity-induced neuroplasticity. Several alternative strategies proposed to bypass the auto-inhibition of 5-HT neurons by targeting other 5-HT receptor subtypes are not discussed here, but have been reviewed recently. These include use of 5-HT4 agonists (Samuels et al., 2016), 5-HT2C or 5-HT7 antagonists (Ramaker and Dulawa, 2017), or 5-HT1B (Nautiyal and Hen, 2017) or 5-HT2B ligands (Quentin et al., 2018) as novel antidepressant strategies that may overcome SSRI resistance (Artigas, 2013).

## The Serotonin System

### Serotonin: Circuitry and Development

The cells responsible for brain 5-HT synthesis uniquely express tryptophan hydroxylase-2 (TPH2), the rate-limiting enzyme for 5-HT synthesis (Walther et al., 2003; Lesch et al., 2012) and are localized in the mesencephalon, pons and medulla oblongata. The majority of 5-HT cells are located in the raphe nuclei in midbrain, in the dorsal, median and caudal raphe sub-regions, which are interconnected [e.g., between dorsal and caudal raphe (Bang et al., 2012)]. The anatomy of the raphe 5-HT projections were characterized initially using histochemical fluorescence (Ungerstedt, 1971), and then using labeled with [^3^H]5-HT, and verified using unilateral injection of the selective serotonin neurotoxin 5,7-dihydroxytryptamine (Azmitia and Segal, 1978; Jacobs and Azmitia, 1992). The rostral dorsal (DR) and median (MR) raphe nuclei project 5-HT fibers via the median forebrain bundle to forebrain areas, while the caudal raphe nuclei innervate cerebellar and spinal targets (Gaspar and Lillesaar, 2012; Maddaloni et al., 2017). Thus, virtually the entire central nervous system receives 5-HT input (Jacobs and Azmitia, 1992). Serotonin neurons within the DR are functionally heterogeneous (Calizo et al., 2011; Okaty et al., 2015; Fernandez et al., 2016) and show diverse projections. Fiber tracing has revealed that individual 5-HT neurons are highly branched and send input to multiple forebrain structures (Gagnon and Parent, 2014). Retrograde tracing using retrobeads indicates that DR subregions may preferentially project to different targets (Waselus et al., 2006) and global mapping of 5-HT projections using viral retrograde tracing has revealed two main projection subtypes: from ventral DR to anterior cortical regions, and from dorsal DR to subcortical regions (Ren et al., 2018). Interestingly, these 5-HT pathways have opposing effects on anxiety and depression phenotypes. In particular, the 5-HT pathway projecting to the amygdala was activated by reward and punishment and promoted anxiety phenotypes, while the frontal cortex projection was activated by reward but inhibited by punishment and promoted an anti-depressed phenotype (Ren et al., 2018). Targeting 5-HT release in the frontal cortex could thus produce a specific and more robust antidepressant effect, avoiding the anxiogenic effects often seen with acute SSRI treatment and thought to involve activation of the amygdala (Arrant et al., 2013). Thus, globally targeting 5-HT using SSRI treatment is likely to activate antagonistic pathways that could contribute to adverse acute effects or to resistance to SSRI treatment.

The raphe nuclei in turn receive inputs from the numerous regions to which the 5-HT neurons project (Pollak Dorocic et al., 2014; Weissbourd et al., 2014; Ren et al., 2018). The majority of projections to the DR are from the hypothalamus, amygdala, medulla and cortex, with the central amygdala sending projections mainly to GABAergic DR interneurons, while the cortical projections are mainly to 5-HT neurons. In contrast, the MR receives sparse input from amygdala, prefrontal cortex (PFC) or other cortical areas, but stronger from hypothalamus and midbrain (Pollak Dorocic et al., 2014). Importantly, DR neurons projecting to anterior cortex receive strongest innervation for cortical regions, while DR cell targeting amygdala receive more innervation from the amygdala (Ren et al., 2018), suggesting a role for feedback regulation of 5-HT projections by the target neurons.

The development of the 5-HT system was addressed in the early 1980s, immunohistochemical studies showed that raphe 5-HT cells are generated from embryonic day (e) e11-15 of gestation in rats and the initial axonal sprouting of 5-HT-containing neurons occurs at e12 (Wallace and Lauder, 1983). By e17, most of the forebrain areas and the frontal part of the neocortex become innervated by 5-HT fibers. The cortical plate is innervated by 5-HT by e18, which forms a deep bundle of fibers sprouting laterally/dorsally within the cortical areas (Jacobs and Azmitia, 1992). The 5-HT innervation terminates in occipital cortex suggesting that 5-HT fibers encircle the brain in a rostro-caudal direction (Wallace and Lauder, 1983). By e21, increases in 5-HT axonal density and terminal formation in subcortical regions are detectable and the latter continues postnatally, resembling the adult brain by post-natal day (p) p3. These early studies were further verified in mice by tracing of GFP-labeled 5-HT neurons through postnatal development (Maddaloni et al., 2017). By p6 increased 5-HT fiber density and terminals are seen in the thalamus, hypothalamus, and cerebellum. At p10-14, 5-HT fibers reach mature levels in all cortex layers and by p21 all terminal fields are fully innervated by 5-HT (Dori et al., 1996; Dinopoulos et al., 1997). Starting from p28, the fibers differentiate to attain an adult morphology (Maddaloni et al., 2017). Using knockout approaches, it has been shown that development of 5-HT projections is dependent on several axonal guidance, planar cell polarity factors (e.g., SLIT1/2, Frizzled3, and Vangl2) (Kiyasova and Gaspar, 2011) and cellular adhesion molecules (e.g., protocadherins) (Katori et al., 2009). In addition to a developmental role, 5-HT is also important for maintenance of 5-HT circuitry in adulthood. Using conditional TPH2GFP knockin mice, 5-HT depletion was found to increase GFP-labeled 5-HT fiber density in the hippocampus, while reducing it in other brain regions, such as the thalamic paraventricular nucleus (Pratelli et al., 2017), implicating 5-HT in development but also maintenance of 5-HT circuitry.

The timing of 5-HT development corresponds with a critical period that has been identified for the development of anxiety and depression phenotypes (Albert et al., 2014; Garcia-Garcia et al., 2014). For example, transient knockout or inhibition of 5-HT1A receptors during the early postnatal-adolescent period results in a persistent anxiety/depression phenotype that is not rescued by gene re-activation in adulthood (Gross et al., 2002; Lo Iacono and Gross, 2008; Donaldson et al., 2014; Garcia-Garcia et al., 2017b). More recently it has been shown that early life manipulation of the 5-HT system, including early life SSRI treatment, alters 5-HT innervation and neural circuitry in adult and impacts adult behavior (Gaspar et al., 2003; Gingrich et al., 2017; Teissier et al., 2017). In this regard, disruption of 5-HT projections by conditional deletion of protocaderin-alphaC2 results in a mild depression-like phenotype in mice, implicating forebrain 5-HT innervation in behavioral phenotypes (Katori et al., 2009; Chen et al., 2017), and as potential target for new antidepressant treatments (Figure 1). The lack of effect on behavior of adulthood 5-HT1A gene rescue in the forebrain of 5-HT1A knockout mice (Gross et al., 2002) suggests that developmental alterations in 5-HT innervation may confer behavioral phenotypes in adulthood that are more resistant to SSRI treatment, although this remains to be tested in these models.

### Serotonin Dynamics: Synthesis and Reuptake

The differentiation of neuronal progenitors to express serotonergic markers like TPH2 is primarily driven by the transcription factor Pet-1, which is expressed only in 5-HT neurons and directly activates the TPH2 gene (Liu et al., 2010; Jacobsen et al., 2011). The activity of TPH is quite low at birth and reaches 60% of adult levels by p2 in rats (Deguchi and Barchas, 1972). Similarly, 5-HT levels reach 75% of adult values by p2 and maximize by p3 (Bennett and Giarman, 1965). The level of extracellular 5-HT in the brain is tightly controlled by the neuronal uptake system at the presynaptic nerve endings to maintain internal homeostasis. Serotonin transporter proteins (SERT) are expressed at release sites and efficiently transport released 5-HT back into the cell via a high affinity Na^+^/Cl^-^ dependent active transport system (Amara and Kuhar, 1993). These high affinity transporters are the targets of many antidepressant compounds. Imipramine and related tricyclic compounds were shown to have antidepressant activity (Lehmann et al., 1958) and then found to inhibit both SERT and the norepinephrine transporter (NET) resulting in a longer half-life of the neurotransmitter in the synaptic cleft (Pletscher, 1991). These and other observations led to the hypothesis that the reduced activity of monoamine systems, like NE and/or 5-HT are associated with depression (Schildkraut, 1965; Coppen, 1967).

### 5-HT Synaptic Contacts and Neuroplasticity

Several modes of 5-HT neurotransmission have been described. Classically, 5-HT neurons arising from the midbrain raphe nuclei are thought to project throughout the brain form direct synapses with target neurons. Interestingly, 5-HT is also released non-synaptically from varicosities (Descarries et al., 1975), a diffusion-based neurotransmission termed “volume transmission” (Fuxe et al., 2007). The 5-HT in an extra-synaptic space preferentially modulates the activity of excitatory/inhibitory synapses, in contrast to its neurotransmitter functions at dendrites or cell bodies (De-Miguel and Trueta, 2005; Kiss, 2008). To finely modulate the activity of excitatory and inhibitory neurons, 5-HT projections form close contacts with these cells, or synaptic triads, as detected by electron microscopy in rodent (Ciranna, 2006). Belmer et al. (2016, 2017) have mapped changes in 5-HTergic axonal density and the formation of triadic connectivity within different corticolimbic regions. They detected 5-HTT+ varicosities in close proximity to presynaptic excitatory and inhibitory nerve terminals. Asymmetrical synapses/excitatory triads were identified in cortical and hippocampal areas, while symmetrical synapses/inhibitory triads were mainly located in subcortical areas. These results suggest that 5-HT projections may preferentially target excitatory vs. inhibitory neurotransmission, depending on the region. The balance between 5-HT regulation of glutamate vs. GABA neurons in the prefrontal cortex is postulated to account for behavioral phenotypes observed upon reduction or activation of 5-HT neurotransmission in transgenic mouse models. Our model postulates that the behavioral phenotype shifts as 5-HT activity increases from none (anxious/aggressive) to low (anxious/depressed) to high (anxious, not depressed) in part due to a dose-dependent shift in 5-HT targeting from glutamate to GABA neurons in the PFC (Albert et al., 2014). The role of 5-HT triads in behavior remains unclear, and the presence of 5-HT triads in human subjects remains to be addressed.

There is evidence that environmental stress can modify the activity of the 5-HT system in a region-specific manner. For example, acute exposure of rats to swim stress increases 5-HT in the striatum but decreases it in the lateral septum and amygdala (Waselus et al., 2006), with no changes in the cortex and hippocampus (Kirby et al., 1995). Stress-induced regional activation targets different 5-HT neuron populations in the DR, but the mechanisms that trigger this specificity remain unclear. In addition, there is evidence that stress-induced neuroplastic changes may remove or alter 5-HT innervation to modify 5-HT action, which can be reversed by deep brain stimulation (Veerakumar et al., 2014). A salient loss of 5-HT fiber density was found in the orbitofrontal cortex (OFC) in depressed subjects, suggesting that region-specific modifications of 5-HT innervation contribute to the pathology of depression (Rajkowska et al., 2017). Harnessing 5-HT neuroplasticity using deep brain stimulation may provide a new treatment strategy in depressed patients that may be resistant to SSRI treatment due to deficient 5-HT innervation in some forebrain areas (Figure 1). Taken together, alterations in the activity of the 5-HT system, including in 5-HT synthesis, 5-HT innervation, or 5-HT degradation differentially impact the activity of the different brain areas and provide potential targets for antidepressant therapy (Figure 1).

In addition to differences in 5-HT projections, different sub-regions of the raphe nuclei have been associated with depression and anxiety. The caudal DR and MR share similar origins and many projections compared to the rostral DR (Commons, 2016). Increasing evidence is suggesting different functional roles of the rostral DR and caudal DR/MR in anxiety and depression phenotypes. Activation of caudal DR by inescapable shock stress or by CRH injection is associated with depression-like behavior providing stronger support for a pro-depression effect of caudal DR (Hammack et al., 2002). In the post-natal FLX model, reduced activity of the rostral DR was associated with behavioral despair, while hyper-activity of the MR led to anxiety-like behavior (Teissier et al., 2015). A recent paper shows that knockout of CACNA1C L-type calcium channel subunit gene in 5-HT neurons results in behavioral despair associated with increased activity of caudal DR and inhibition of the rostral DR, via 5-HT1A receptor activation (Ehlinger and Commons, 2019). In post-mortem raphe tissue from depressed suicide compared to control brains, TPH2 RNA is increased in mid-caudal DR (Bach-Mizrachi et al., 2008) and 5-HT1A receptors in higher in mid-rostral DR than in caudal DR (Stockmeier et al., 1998; Boldrini et al., 2008), which may suggest reduced 5-HT1A-mediated auto-inhibition of the caudal DR vs. rostral DR. Thus, region-specific activation of the caudal DR/MR and 5-HT1A-induced inhibition of rostral DR appear to associate with depression and possibly anxiety phenotypes.

## 5-HT1A Receptors

### 5-HT1A Autoreceptor and Heteroreceptor Function

**FIGURE 2:**
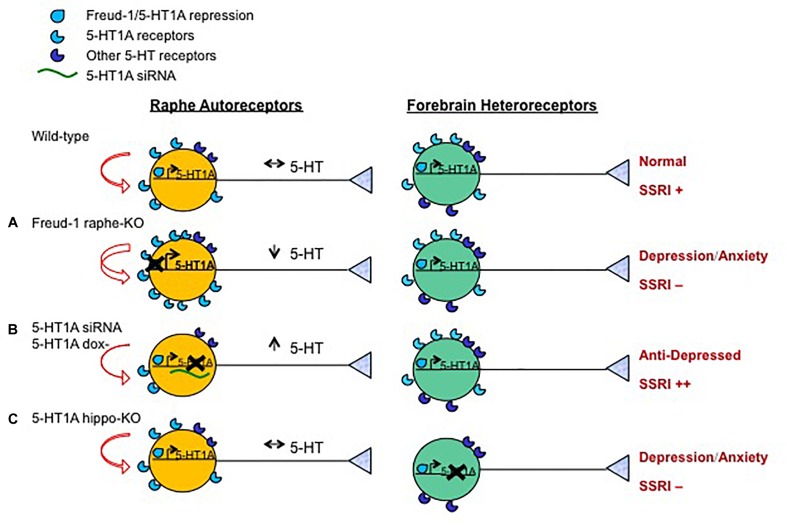
Opposite roles of 5-HT1A autoreceptor vs. heteroreceptors in behavior and SSRI response. Shown are the effects on the serotonin system and behavior in mouse or rat knockout/knockdown (X) genetic models compared to wild-type animals. Models included were generated with: **(A)** Over-expression of 5-HT1A autoreceptors (using knockout of 5-HT1A repressor Freud-1/CC2D1A in adult 5-HT neurons); **(B)** knock-down of 5-HT1A autoreceptors (using raphe-targeted 5-HT1A siRNA (Bortolozzi et al., 2012) or inducible genetic knock-down); or **(C)** loss (using gene knockout) of 5-HT1A heteroreceptors in hippocampal granule cells. The effect of these knockouts on 5-HT1A transcription (right angle arrow) and receptor levels, 5-HT auto-inhibition (curved red arrows), 5-HT neuronal activity (black arrows), depression- or anxiety-like behavior and response to chronic SSRI treatment (++, + or -) are shown. Increasing the expression of 5-HT1A autoreceptors reduces 5-HT activity leading to anxiety and/or depression-like behavior resistant to SSRI treatment (Vahid-Ansari et al., 2017). Knockdown of adult 5-HT1A autoreceptors induces a stress-resilient state and enhances SSRI responsiveness (++) (Richardson-Jones et al., 2010), but can also lead to an anxiogenic response to subchronic SSRI with extensive knockdown (Turcotte-Cardin et al., 2019). The loss of hippocampal granule cell 5-HT1A heteroreceptors leads to depression and anxiety phenotypes and prevents SSRI response (Samuels et al., 2015).

Additional important targets of antidepressant treatment are the 5-HT receptors, particularly the 5-HT1A receptor (Figure 1). A large family of at least 14 distinct receptor subtypes mediates the actions of 5-HT on brain function (Hoyer et al., 2002). Much attention has focused on the 5-HT1A receptor subtype, among the most abundant and widely expressed 5-HT receptors in the brain (Barnes and Sharp, 1999; Beliveau et al., 2017). 5-HT1A receptors have a dual function: as somatodendritic autoreceptors located on 5-HT neurons in the raphe nuclei; and as postsynaptic heteroreceptors, which exist on target non-5-HT neurons in 5-HT projecting areas (Figure 2) (Riad et al., 2001; Albert, 2012; Garcia-Garcia et al., 2014). Serotonin released in the raphe activates 5-HT1A autoreceptors to negatively regulate the firing of the serotonin system (Blier et al., 1998; Albert and Francois, 2010; Garcia-Garcia et al., 2014) (Figure 2), although with greater inhibition in DR compared to MR (Beck et al., 2004). This 5-HT1A-mediated auto-inhibition is often not observed *in vitro*, unless physiological levels of tryptophan are added to support 5-HT synthesis (Liu et al., 2005). Release of serotonin at target neurons activates 5-HT1A heteroreceptors that are most abundantly expressed in the hippocampus, septum, amygdala, and PFC (Albert et al., 1990), where it mediates 5-HT actions on fear, anxiety, stress, and cognitive function (Albert et al., 2014; Garcia-Garcia et al., 2014). In the forebrain, 5-HT1A heteroreceptors are expressed on two antagonistic neuronal populations, to modulate the activity of excitatory glutamatergic pyramidal neurons and inhibitory GABAergic interneurons, in parallel (Celada et al., 2013; Albert et al., 2014). Thus, 5-HT1A receptors both negatively regulate global 5-HT activity and mediate 5-HT responses in target neurons.

The roles of 5-HT1A receptors in brain function have been tested pharmacologically by the administration of 5-HT1A receptor-selective compounds (Fletcher et al., 1996). However, these compounds do not discriminate between 5-HT1A auto- or heteroreceptors, but target all 5-HT1A receptors. They include the agonist 8-hydroxy-2-(di-n-propylamino)tetralin (8OH-DPAT), which is selective for 5-HT1A receptors, but can activate 5-HT7 receptors with 10-fold lower affinity. The antagonist WAY100635 is highly specific in blocking 5-HT1A receptors, but activates dopamine-D4 receptors with 10-fold lower potency (Chemel et al., 2006). Systemic administration of 8-OH-DPAT produces acute hyperphagia, hypothermia, and an anxiolytic effect in rodents. The behavioral and physiological effects of 8OH-DPAT are blocked by pretreatment with the 5-HT1A antagonist, WAY 100635. In clinical trials, 5-HT1A partial agonists, such as buspirone, are currently used as anxiolytics (Lucki, 1996). Buspirone and other 5-HT1A receptor partial agonists and antagonists are also reported to enhance the therapeutic effects of antidepressants (Blier and Ward, 2003), as seen in the Sequenced Treatment Alternatives to Relieve Depression (STAR^∗^D) study (Trivedi et al., 2006a) (Figure 1). These anxiolytic compounds appear to trigger distinct signaling pathways from full agonists like 5-HT or 8OH-DPAT (Albert et al., 1999; Pauwels and Colpaert, 2003), which may contribute to their preferential actions on anxiety and to augment SSRI actions, but this remains to be clarified.

### 5-HT1A Receptor Signaling

The 5-HT1A receptor signals via inhibitory G proteins (Gi/Go) to reduce neuronal excitability and inhibit firing rate. The 5-HT1A auto- and hetero-receptors have the same intronless coding sequence and share canonical Gi/Go signaling pathways including: inhibition of adenylyl cyclase to reduce PKA activity; opening of G-protein inward rectifying potassium (GIRK) channels to hyperpolarize membrane potential; and inhibition of voltage-gated calcium channels (Ca^2+^) to reduce calcium influx and calcium-calmodulin kinase activity (Raymond et al., 1999; Albert and Vahid-Ansari, 2018). Nevertheless, they appear to signal differently to G proteins and to tyrosine kinase effectors such as ERK1/2 and Akt-GSK3 (Polter and Li, 2010; Albert and Vahid-Ansari, 2018). For example, 5-HT1A autoreceptors couple to inhibition of ERK1/2 activation in raphe RN46A cells (Kushwaha and Albert, 2005), while the hippocampal 5-HT1A heteroreceptor activates ERK1/2 via a PKC-mediated pathway (Adayev et al., 1999; Mogha et al., 2012). The latter pathway is implicated in 5-HT-induced hippocampal synaptogenesis in development (Mogha et al., 2012), while the former may participate in long-term 5-HT1A-induced auto-inhibition of raphe function. Recent transgenic models have implicated hippocampal 5-HT1A-Gi2 signaling and Akt-GSK3β in fluoxetine-induced anti depressant actions in mice (Talbot et al., 2010; Polter et al., 2012). Importantly, global 5-HT1A knockout (Santarelli et al., 2003) or specific deletion of the gene in hippocampal dentate gyrus granule cells (Samuels et al., 2015) prevents fluoxetine-induced hippocampal neurogenesis and anti depressant and anti-anxiety actions, implicating hippocampal 5-HT1A receptors in fluoxetine action (Figure 2). Taken together, 5-HT1A autoreceptors and heteroreceptors signal to diverse, sometimes opposing pathways to mediate the acute and sustained effects of the serotonin system on anxiety and depression (Albert and Vahid-Ansari, 2018). Identifying biased 5-HT1A ligands that target autoreceptor vs. heteroreceptor signaling may provide useful tools to separately target these receptors that have opposite effects on depression and SSRI response (Figure 2) (Assie et al., 2010; Becker et al., 2016).

## Serotonin Projections and Behavior

### Optogenetic and Chemogenetic Studies

The 5-HT system plays important roles in physiological and behavioral function. Despite the large body of studies implicating functional perturbations in this system in a variety of behaviors, the role of specific 5-HT projections from different parts of the raphe remains unclear. Recently, optogenetic studies directly activating 5-HT neurons have revealed novel and sometimes opposing roles for DR stimulation in behavior. For example, Liu and coworkers showed that the acute activation of 5-HT cells in DR results in reinforcement (Liu et al., 2014), while others found a role for this pathway in patience for delayed reward, with no role in reinforcement (Fonseca et al., 2015). Similarly, in one study acute photostimulation of DR 5-HT cells reversibly decreased mechanosensory responses in behaving mice (Dugue et al., 2014), while another showed that phasic optogenetic activation of DR 5-HT neurons produces a transient inhibition in locomotion, but also a persistent increase after chronic stimulation (Correia et al., 2017). Optogenetic stimulation of 5-HT neurons in the MR produced an anxiogenic effect in the elevated plus maze while no behavior changes were found when stimulating the DR (Ohmura et al., 2014). However, stimulating DR projections to the dorsal bed nucleus of the stria terminalis (BNST) to activate inhibitory 5-HT1A receptors was anxiolytic in the elevated plus maze test, and inhibition of those 5-HT axons was instead anxiogenic (Garcia-Garcia et al., 2017a). Oppositely, optogenetic activation at higher power of a DR projection to the ventral BNST activates (via excitatory 5-HT2C receptors) an anxiogenic CRH neurons that silence anxiolytic BNST output neurons (Marcinkiewcz et al., 2016). This latter circuit appears mediate an acute SSRI-induced anxiogenic response. It is likely that 5-HT input to different neuronal populations (e.g., glutamatergic vs. GABAergic) via different receptors (e.g., 5-HT1A vs. 5-HT2) can have opposing effects on behavior (Albert et al., 2014). Thus, targeting the 5-HT system using SSRIs may trigger antagonist neural circuits in some individuals that minimize their efficacy as anti-anxiety or antidepressant treatments, or even lead to adverse effects like anxiogenesis. In these cases, augmentation of SSRI with 5-HT1A partial agonists may enhance the antidepressant response by modulating receptor-specific stimulation by 5-HT.

With regard to the role of different subsets of 5-HT neurons in anxiety- or depression-like behavior, a recent report in mice shows that activating 5-HT neurons of the DR with different projections can have opposite effects on anxiety (Ren et al., 2018). Chemogenetic activation of DR 5-HT neurons projecting to the amygdala, BNST and paraventricular hypothalamus promoted anxiety-like behavior. These 5-HT neurons in the DR express SERT but not vGLUT3 and conditional knockout of TPH2 in this pathway revealed a prodepressant effect of this pathway. In contrast, a distinct population of 5-HT neurons projecting to the anterior cortical areas including the OFC, improved coping behavior in the face of challenge, and had an anti-anxiety effect. The role of 5-HT in these actions was verified by conditional knockout of TPH2 in this pathway. Interestingly, in human depressed subjects a deficiency in 5-HT axon length in OFC Layer VI has been reported (Rajkowska et al., 2017). In mice, the OFC-projecting 5-HT cells co-express SERT and VGLUT3 and are located in the ventral DR (Ren et al., 2018). In agreement, specific inhibition of the DR in mice had prodepressant actions, while MR inhibition had the opposite effect (Teissier et al., 2015). Chemogenetic stimulation of the raphe induced antidepressant and anti-anxiety effects, which were blunted in a depression model (Teissier et al., 2015). Similarly, optogenetic stimulation of the MR also induced anxiety-like behavior (Ohmura et al., 2014). These studies lead to the hypothesis that the heterogeneity of 5-HT neuron projections in the DR and MR is associated with distinct behavioral outcomes, and their activity can be modified by changes in activity associated with depression (Teissier et al., 2015). New clinical approaches involving stimulation of discrete brain regions implicated in depression may activate the appropriate 5-HT circuits to induce stable remission, particularly in the presence of concurrent SSRI treatment (Figure 1). In this regard, a recent prospective study has used deep transcranial magnetic stimulation (dTMS) in SSRI-resistant patients and show enhanced response with SSRI compared to dTMS alone (Tendler et al., 2018).

### Serotonin and Neuroplasticity

The adult brain can adapt to environmental and internal stimuli with structural and functional changes known as plasticity and the 5-HT system appears to enhance neuroplasticity of target brain areas (Azmitia, 1999; Batsikadze et al., 2013; Castren and Hen, 2013; Kraus et al., 2017). For example, neuroplasticity following monocular deprivation in adult rats is restored by chronic SSRI treatment (Maya Vetencourt et al., 2008). In human depressed post-mortem brain compared to controls, a lower density of SERT+ 5-HT axons is seen in orbitofrontal cortex (Rajkowska et al., 2017). Conversely, excessive 5-HT during development is associated with dystrophic 5-HT projections in human autism (Daubert and Condron, 2010; Azmitia et al., 2011a,b) and defects in cortical pyramidal and interneuron migration, as seen in 5-HTT–/– mice (Riccio et al., 2009; Riccio et al., 2011). In rodents and humans, chronic SSRI treatment increases the expression in hippocampus and cortex of neurotrophins like brain-derived neurotrophic factor (BDNF), in part via activation of the transcription factor, CREB (D’Sa and Duman, 2002) to mediate behavioral improvement (Jin et al., 2017). Reductions in hippocampal BDNF are seen in human depression (Chen et al., 2001) and associated with the BDNF-Val66Met and 5-HTTLPR risk alleles and reduced 5-HT1A receptor function (Chen et al., 2006; Homberg et al., 2014). Yet, the effect of these polymorphisms on response to SSRI treatment in human depression remains controversial (Domschke et al., 2010).

The 5-HT system also has a modulatory effect on long-term synaptic plasticity of glutamatergic neurotransmission underlying LTP and/or LTD in learning and memory, with no changes in glutamate level (Fernandez et al., 2017). In the amygdala, 5-HT release and activation of 5-HT1A heteroreceptors induced a reduction in excitatory synaptic transmission followed by a 5-HT4 receptor-mediated potentiation (Huang and Kandel, 2007). Conditioned fear stress increases 5-HT levels in the amygdala and mediates LTP via 5-HT1A receptor activation (Yokoyama et al., 2005; Johansen et al., 2011). 5-HT1A receptor activation reduces EPSPs in several brain regions by activities such as down-regulation of NR2B receptors in cortical pyramidal neurons (Yuen et al., 2005) or reductions of AMPA currents and surface expression of GluR2/3 receptors (Schiapparelli et al., 2005). Thus, 5-HT1A heteroreceptors can alter plasticity through a variety of mechanisms including regulation of glutamate receptors, synapse formation, alterations in 5-HT projections. The ability to target these post-synaptic signaling mechanisms could enhance response to SSRI treatment (Figure 1).

In addition, 5-HT neurons themselves undergo neuroplasticity in response to chronic antidepressant treatment. In particular, chronic deep brain stimulation of the prefrontal cortex increases social interaction in chronic social defeat mice. Cortical stimulation was associated with recovery of 5-HT neuron firing activity, reduction of 5-HT neuron dendritic length and branching, increased glutamatergic synapses in the DR, and recovery of 5-HT synaptic density and/or size in the PFC, hippocampus and amygdala (Veerakumar et al., 2014). Thus chronic brain stimulation can induce 5-HT plasticity both in the raphe and in its projections to target regions.

### 5-HT1A-Mediated Neurogenesis and Neuroplasticity

In addition to regulating neuroplasticity, 5-HT1A receptors have been implicated in adult hippocampal neurogenesis (Santarelli et al., 2003; Banasr et al., 2004; Klempin et al., 2010). Direct activation of 5-HT1A receptors using 8OH-DPAT increases progenitor cell proliferation, which is blocked by 5-HT1A antagonist WAY100635 (Banasr et al., 2004; Klempin et al., 2010) or an inhibitor of ERK1/2 signaling (Cai et al., 2019). The effects of chronic SSRI treatment on hippocampal neurogenesis and anxiety- or depression-like behavior are blocked using 5-HT1A antagonists (Klempin et al., 2010) or by knockout of 5-HT1A receptors, globally or specifically on granule cells of the hippocampus (Santarelli et al., 2003; Samuels et al., 2015) (Figure 2). These findings suggest that the behavioral effects of chronic SSRI treatment are mediated by stimulation of hippocampal neurogenesis, which requires 5-HT1A heteroreceptors. On the other hand, mice with over-expression of 5-HT1A heteroreceptors show increased adult neurogenesis (in females but not males) (Noto et al., 2016), suggesting that 5-HT1A receptor levels can drive increase in hippocampal neurogenesis. However, knockout models of loss of 5-HT1A receptors or of 5-HT did not alter basal neurogenesis (Santarelli et al., 2003; Diaz et al., 2013). In addition, the role of 5-HT1A receptors in adult hippocampal neurogenesis in humans has not been addressed. The extent of hippocampal neurogenesis in adult humans, though supported by solid evidence, remains difficult to assess (Kempermann et al., 2018). Directly targeting 5-HT1A-induced neurogenesis could bypass resistance to SSRIs associated with reduced activity of 5-HT neurons (e.g., due to increase 5-HT1A autoreceptors, Figure 2).

Thus, the therapeutic action of antidepressants is dependent on a balanced activity of 5-HT1A auto vs. heteroreceptors (Samuels et al., 2015) (Figure 2). Activation of 5-HT1A autoreceptors reduces 5-HT activity and response to SSRI treatment. Activation of 5-HT1A heteroreceptors stimulates hippocampal neurogenesis and regulates dendritic maturation in the hippocampus and frontal cortex associated with the antidepressant response (Samuels et al., 2015). Consistent with roles in neuroplasticity and neurogenesis, levels of 5-HT1A auto- and heteroreceptors appear to correlate oppositely with cortical gray matter thickness. Multimodal imaging studies show that in MDD patients, an increase in raphe 5-HT1A binding potential is correlated with reduced cortical thickness values and fewer 5-HT tracts projecting to the cortex, while increased terminal 5-HT1A receptors correlate with increased gray matter volume in several cortical and hippocampal regions (Kraus et al., 2012; Zanderigo et al., 2018). Therefore, higher activity of 5-HT1A autoreceptors residing on serotoninergic raphe cells puts the brakes on 5-HT neurotransmission in target areas and affects their synaptic plasticity.

## Serotonin, Antidepressants, and Depression

### Clinical Studies

Several lines of evidence have implicated reduced 5-HT as a key risk factor for major depression. The current first-line therapy for major depression targets the 5-HT system (Trivedi et al., 2006b). In particular, selective 5-HT reuptake inhibitors (SSRIs), such as fluoxetine or citalopram, specifically block SERT to selectively increase 5-HT neurotransmission. This selectivity for 5-HT results in less severe adverse effects compared to tricyclic antidepressants like imipramine that also target noradrenalin reuptake (Cipriani et al., 2016, 2018) and can block alpha1-adrenergic receptors, inducing orthostatic hypotension in some patients (Glassman, 1984). Chronic treatment with SSRI was shown to be effective in major depression, anxiety, and several other mood disorders, implicating serotonin (Charney et al., 1990). Acute tryptophan depletion to acutely reduce 5-HT, also supports a role for decreased 5-HT in depression, or at least in the relapse of recovered depressed patients (Leyton et al., 1997; Delgado et al., 1999; Young and Leyton, 2002; Jans et al., 2007). 5-HT and its metabolites are reduced in the cerebrospinal fluid of depressed patients and especially of depressed suicide victims (Asberg and Traskman, 1981; Asberg, 1997; Mann and Malone, 1997; Moberg et al., 2011). Reductions cortical 5-HT receptor levels are also seen in PET imaging studies of living depressed patients and in post-mortem studies (Yatham et al., 2000; Mintun et al., 2004; Berney et al., 2008; Savitz and Drevets, 2009; Hesselgrave and Parsey, 2013; An et al., 2016). Several studies have found that normal women have significantly higher 5-HT1A receptor (Parsey et al., 2002; Jovanovic et al., 2008) and lower 5-HTT binding potentials (Jovanovic et al., 2008; Underwood et al., 2018) than men, in raphe and several cortical regions. By contrast in male but not female MDD, 5-HT1A autoreceptors were increased compared to controls (Kaufman et al., 2015), while females showed reduced PFC 5-HT1A receptors (Szewczyk et al., 2009). In post-mortem studies, decreases in cortical 5-HT1A receptor RNA and binding site levels are observed (Lopez-Figueroa et al., 2004; Stockmeier et al., 2009; Szewczyk et al., 2009), but in some areas increases are seen (Anisman et al., 2008). Oppositely, increased levels of 5-HT1A autoreceptor binding have been reported in the DR, particularly in the rostral DR (Stockmeier et al., 1998; Boldrini et al., 2008). Similarly in PET imaging studies, reductions in cortical 5-HT1A heteroreceptors and increases in raphe 5-HT1A autoreceptors have been found in depressed subjects compared to controls (Savitz et al., 2009; Hesselgrave and Parsey, 2013; Kautzky et al., 2017; Milak et al., 2018). These results suggest a reduced activity of the 5-HT system driven by increased 5-HT1A autoreceptors and/or reduced 5-HT1A heteroreceptors may predispose to MDD. In agreement, functional variants affecting gene expression in the 5-HT system, such as the 5-HTTLPR and 5-HT1A rs6295 polymorphisms have been implicated in disease susceptibility and response to antidepressants (Serretti et al., 2007; Le Francois et al., 2008; Newman-Tancredi and Albert, 2012; Pettitt, 2015). However, these markers alone are not robust enough to predict response to SSRI treatment (Kato et al., 2015), which remains insufficient. The STAR^∗^D study showed that only one-third of patients given the SSRI citalopram as first-line treatment achieved remission and that about 10–15% more responded to combination therapy (Trivedi et al., 2006b). Therefore, in many patients, targeting the serotonin system or other monoamine systems is insufficient for benefit. Patients who fail two or three types of treatments are classified as treatment-resistant (Rush et al., 2009). Identifying animal models that can be used to address mechanisms of treatment resistance and how to overcome it remains a major challenge (O’Leary et al., 2014; Willner and Belzung, 2015).

### Rodent Models of SSRI Resistance

Several transgenic and knockout mouse models indicate the role of dis-inhibition of the 5-HT system in response to SSRI treatment (Figure 2). A common theme is that 5-HT1A autoreceptor-mediated inhibition of 5-HT neurons prevents behavioral responses to SSRI treatment (Artigas et al., 1996; Blier and Ward, 2003). First, the time course of 5-HT1A autoreceptor desensitization follows the latency for clinical response to SSRI and other antidepressants, suggesting that reduced 5-HT auto-inhibition is required for SSRI response (Blier and Ward, 2003). Second, pharmacological inhibition [e.g., using pindolol (Blier and Ward, 2003)] or inducible repression of 5-HT1A autoreceptors in adult 5-HT neurons was shown to enhance and accelerate response to SSRI treatment (Richardson-Jones et al., 2010). Similarly, acute down-regulation of 5-HT1A autoreceptors using 5-HT1A-siRNA targeted to 5-HT neurons induces an acute antidepressant response in rats (Bortolozzi et al., 2012). However, mice with more extensive knockdown (in 90% of 5-HT neurons) of 5-HT1A autoreceptors results in an anxiogenic response to sub-chronic (9 d) SSRI treatment in both sexes, which may be due to hyper-activation of the 5-HT system (Turcotte-Cardin et al., 2019). Third, targeted gene deletion of a repressor of the 5-HT1A receptor gene (Freud-1) in adult 5-HT neurons to up-regulate 5-HT1A autoreceptors, reduces 5-HT neuronal activity and results in a fluoxetine-resistant anxiety and depression phenotype in both male and female mice (Vahid-Ansari et al., 2017). Taken together, these different rodent models implicate 5-HT1A autoreceptors in SSRI resistance (Figure 2).

In contrast to 5-HT1A autoreceptors, specific deletion of hippocampal granule cell 5-HT1A heteroreceptors prevents SSRI/CC2D1A-induced hippocampal neurogenesis and antidepressant actions (Samuels et al., 2015) (Figure 2). Similarly, mice with global knockout of 5-HT1A receptors are also resistant to SSRI treatment (Santarelli et al., 2003). However, chronic desipramine, which targets 5-HT and noradrenalin transporters, reversed the behavioral phenotype (Santarelli et al., 2003). Thus, while 5-HT1A autoreceptors inhibit SSRI response, 5-HT1A heteroreceptors in the hippocampus are required for SSRI actions. Antidepressants that target a different system such as desipramine, can still mediate the behavioral response. This provides empirical support for the concept that patients that do not respond to SSRIs should be switched to a different class of antidepressant (Blier, 2014). However, patients do not entirely lack 5-HT1A receptors and may still respond to augmentation with drugs that inhibit or desensitize 5-HT1A autoreceptors, such as buspirone or pindolol (Artigas et al., 2018). Despite promising results showing that pindolol augmentation could accelerate and enhance SSRI response patients (Artigas et al., 1994; Blier and Bergeron, 1995; Portella et al., 2009), not all studies have shown benefit, especially in SSRI-resistant cohorts (McAskill et al., 1998; Berman et al., 1999; Perez et al., 1999). This could reflect inadequate dosing (Martinez et al., 2000), non-selective effects of pindolol on both 5-HT1A auto- and heteroreceptors (Newman-Tancredi et al., 2001), or antagonism of beta-adrenergic receptors. Partial block of postsynaptic 5-HT1A receptors by pindolol may prevent pindolol’s benefits due to block of 5-HT1A autoreceptors, emphasizing the need for compounds with higher selectivity to block presynaptic or activate post-synaptic 5-HT1A receptors (Garcia-Garcia et al., 2014; Vidal et al., 2018).

Given the sex differences in depression prevalence and 5-HT1A receptor levels in humans, a few studies have addressed sex differences in transgenic models of 5-HT1A receptor regulation. For example, knockout of the HTR1A repressor Deaf1 results in increased 5-HT1A autoreceptor expression but greater functional uncoupling in females, and a sex- and test-dependent anxiety phenotype (Luckhart et al., 2016). Similarly knockout in adult 5-HT neurons of MeCP2, an enhancer Deaf1 repressor activity, also increased 5-HT1A receptor expression, resulting in a similar sex- and test-dependent anxiety and depression phenotypes (Philippe et al., 2018). In contrast, knockout in 5-HT neurons of the stronger repressor Freud-1 resulted in a consistent anxiety and depression phenotype in all tests (Vahid-Ansari et al., 2017). The extent of up-regulation of 5-HT1A autoreceptors in the latter model may overwhelm endogenous sex-dependent differences to result in SSRI-resistant anxiety and depression. Similarly, over-expression of 5-HT1A heteroreceptors mainly in cortical regions reduced depression-like behavior in males but not females, yet both responded equally to SSRI treatment (Gunther et al., 2011).

Another mouse model of SSRI resistance is the BDNF Val66Met mouse, in which the human BDNF gene and polymorphism was knocked-in to the mouse locus, which phenocopies humans with this polymorphism (Chen et al., 2006). The BDNF (Met) mice display an anxiety-like phenotype that is resistant to chronic FLX (Chen et al., 2006), ketamine (Liu et al., 2012) or voluntary exercise (Ieraci et al., 2016). However, the heterozygous BDNF (+/Met) mice that fail to respond to FLX responded to chronic desipramine (Yu et al., 2012). Clinically, antidepressants targeting multiple neurotransmitter systems, such as imipramine that targets 5-HT and noradrenalin, appear more effective than SSRIs, but may also have lower acceptability and compliance rates (Cipriani et al., 2016, 2018). An alternative strategy to directly reverse BDNF deficiency is intranasal administration of a viral brain-permeant BDNF construct, which reversed the depression phenotype in mice following chronic mild stress (Ma et al., 2016).

Taken together, these animal model studies implicate alterations in 5-HT and BDNF in resistance to SSRI treatment. Patients with polymorphisms (5-HTTLPR, 5-HT1A rs6295, BDNF Val66Met) that alter the function of key genes in these systems may be more resistant to SSRI treatment, but responsive to treatments that target other systems (noradrenalin) or that can augment 5-HT activity to permit SSRI action (Figure 1). Still, there is no reliable marker to predict which treatment option would be best.

## How to Improve Treatment for Major Depression?

### Markers for SSRI Response

Although SSRIs remain the first-line treatment for MDD, due to the low remission rate (30% of patients remit) and long latency, many patients must be switched to other antidepressants, or given augmentation to try to enhance SSRI response. In the STAR^∗^D study, patients not responding to SSRI were switched to another antidepressant or augmented with add-on treatments, leading to around 50–60% remission rate and 40% treatment resistant patients failing to respond to two chronically used common antidepressants (Rush et al., 2009). Citalopram non-responders switched to another SSRI (27% response) or a different class of antidepressant (25–6% response) had similar response rates (Figure 1). Thus, there was no clear indication of which treatment would be best for a given patient who fails to respond or remit on SSRI treatment.

Resistance to SSRIs appears to be associated with genetic polymorphisms in 5-HT-related genes, like the 5-HTT (SLC6A4) and HTR1A genes. For example, the HTR1A rs6295 polymorphism has been associated with depression, suicide and SSRI resistance (Le Francois et al., 2008). Other gene polymorphisms including BDNF (rs10501087 and Val66Met rs6265), 5HT2A receptor (rs7997012) and CREB1 (rs7569963) have been shown to interact with each other in predicting SSRI resistance (Kautzky et al., 2015). Among those, BDNF rs6265 has been the focus of many studies with the most robust association with SSRI response (Niitsu et al., 2013). More recently, progress has been made in using functional MRI (fMRI) of resting state functional connectivity to identify depression subtypes and subjects that are resistant to SSRI or cognitive-behavioral therapy (Dunlop et al., 2017), and responsive to targeted repetitive transcranial magnetic stimulation (rTMS) (Drysdale et al., 2017). In a small cohort of treatment-resistant patients, deep brain stimulation (DBS) targeting a hub of the depression circuitry [subgenual cingulate gyrus (Cg25)] improved negative mood and depressive illness (Kennedy et al., 2011). However, stimulation of the subcallosal cingulate white matter was ineffective (Holtzheimer et al., 2017), suggesting that further study is required.

Recently, altered left-right asymmetry in electro-encephalogram (EEG) has been suggested to predict response to the SSRI escitalopram, with elevated alpha and reduced delta power in the right hemisphere predicting non-response (Baskaran et al., 2018). Interestingly, the HTR1A rs6295 risk polymorphism is also associated with EEG asymmetry with greater right frontal activity (Bismark et al., 2010), and has also been associated with resistance to SSRI treatment. The EEG approach has also been applied to detect increased 5-HT1A autoreceptor activity in response to buspirone in a small cohort of depressed compared to normal controls (McAllister-Williams et al., 2014), and might provide a non-invasive marker of resistance to SSRI treatment due to increase in 5-HT1A autoreceptor function.

### Ketamine and Serotonin

In clinical research studies, there has been a large interest in the effects of single sub-anesthetic dose of infused ketamine (0.5 mg/kg, in 40 min) in treatment resistant depression patients (Zarate et al., 2006, 2012). However, after single infusion the antidepressant response was not sustained. Further studies showed that repeated twice-weekly ketamine treatment maintains the antidepressant response for up to 2 weeks (Singh et al., 2016). In control male volunteers, ketamine-induced interneuron inhibition is associated with increased synaptic glutamate in the anterior cingulate cortex (Stone et al., 2012). Therefore, region-dependent effects of ketamine may be associated with the efficacy of ketamine to treat TRD.

The exact mechanisms of ketamine action remain unclear, with the recent finding that ketamine metabolites without NMDA blocking activity exert antidepressant actions (Zanos et al., 2016). Low dose ketamine is thought to block NMDA receptors preferentially on GABAergic interneurons to enhance AMPA receptor signaling to release to mTOR to increase synaptogenesis in prefrontal cortical pyramidal neurons (Zanos and Gould, 2018). Ketamine metabolites appear to directly activate AMPA signaling to trigger this pathway to induce the rapid (1 h) antidepressant actions of ketamine. Recent studies in rodents implicate the 5-HT system in ketamine action (Nishitani et al., 2014; du Jardin et al., 2016; Fukumoto et al., 2016; Pham et al., 2017). In particular, ketamine increases 5-HT levels in prefrontal cortex (Pham et al., 2017), and mediates antidepressant-like activity in the forced swimming test assayed 24 h post-treatment via activation of PFC 5-HT1A heteroreceptors (Fukumoto et al., 2016, 2018). This sustained antidepressant-like activity requires activation of raphe AMPA receptors that recruits the prefrontal cortex neural circuit (Pham et al., 2017; Pham and Gardier, 2019). Furthermore, AMPA receptor-dependent 5-HT release and activation of PFC 5-HT1A receptors mediates the antidepressant actions of an mGlu2/3 receptor antagonist in the forced swim test (24 h), via 5-HT1A signaling to Akt-mTOR (Fukumoto et al., 2016, 2018). Given the role of 5-HT in the sustained actions of ketamine and mGluR2/3 antagonists, it would be interesting to test their efficacy in SSRI-resistant mouse models.

## Conclusion

A multi-factorial disease like depression cannot always be managed using a one-dimensional strategy, such as targeting the 5-HT system. While SSRIs may be effective in some patients initially, epigenetic and environmental factors can prevent or gradually erode the response to treatment (Albert and Lemonde, 2004). This review illustrates how region-specific activation of 5-HT mechanisms including synthesis, 5-HT neuronal activity, 5-HT neuroplasticity, and 5-HT-induced hippocampal neurogenesis could augment SSRI response (Figure 1). This activation could be mediated by targeted brain stimulation to regions with abnormal brain activity in imaging (Drysdale et al., 2017; Dunlop et al., 2017) or EEG analysis (Baskaran et al., 2018). Furthermore, strategies that overcome 5-HT1A-mediated autoinhibition of 5-HT activity may also overcome resistance to SSRIs, and could include augmentation with 5-HT1A partial agonists or switching to drugs that target additional or other systems such as noradrenalin, dopamine or glutamate (Figure 1). However, there remain no clear biomarkers that can predict resistance to antidepressants, or whether augmentation or switching antidepressants is best. By testing responses in SSRI resistant models that mimic or phenocopy human genetic polymorphisms such as 5-HT1A autoreceptor over-expression or BDNF Val66Met genotype, progress is being made to understand the mechanistic underpinnings of SSRI resistance. In addition to providing potential markers, such as risk polymorphisms or imaging changes, a mechanistic understanding is providing novel ways of targeting the 5-HT system, such as SSRI-conjugated siRNAs to target 5-HT neurons (Bortolozzi et al., 2012). Ultimately, non-invasive methods to identify treatment-resistance may lead to novel strategies that combine brain stimulation with neurotransmitter modulation to accelerate and enhance antidepressant response (Tendler et al., 2018).

## Author Contributions

FV-A and PA conceived the content. FV-A, AZ, and MZ wrote the first draft. PA revised the final draft. All authors approved the final draft.

## Conflict of Interest Statement

The authors declare that the research was conducted in the absence of any commercial or financial relationships that could be construed as a potential conflict of interest.
